# Matrix scaffolds for endometrium-derived organoid models

**DOI:** 10.3389/fendo.2023.1240064

**Published:** 2023-08-10

**Authors:** Silke De Vriendt, Celia Mesias Casares, Susana Rocha, Hugo Vankelecom

**Affiliations:** ^1^ Laboratory of Tissue Plasticity in Health and Disease, Cluster of Stem Cell and Developmental Biology, Department of Development and Regeneration, Katholieke Universiteit (KU) Leuven, Leuven, Belgium; ^2^ Molecular Imaging and Photonics, Department of Chemistry, Katholieke Universiteit (KU) Leuven, Heverlee, Belgium

**Keywords:** endometrium, organoid, matrix scaffold, extracellular matrix, hydrogel

## Abstract

The uterus-lining endometrium is essential to mammalian reproduction, receiving and accommodating the embryo for proper development. Despite its key role, mechanisms underlying endometrial biology (menstrual cycling, embryo interaction) and disease are not well understood. Its hidden location in the womb, and thereby-associated lack of suitable research models, contribute to this knowledge gap. Recently, 3D organoid models have been developed from both healthy and diseased endometrium. These organoids closely recapitulate the tissue’s epithelium phenotype and (patho)biology, including *in vitro* reproduction of the menstrual cycle. Typically, organoids are grown in a scaffold made of surrogate tissue extracellular matrix (ECM), with mouse tumor basement membrane extracts being the most commonly used. However, important limitations apply including their lack of standardization and xeno-derivation which strongly hinder clinical translation. Therefore, researchers are actively seeking better alternatives including fully defined matrices for faithful and efficient growth of organoids. Here, we summarize the state-of-the-art regarding matrix scaffolds to grow endometrium-derived organoids as well as more advanced organoid-based 3D models. We discuss remaining shortcomings and challenges to advance endometrial organoids toward defined and standardized tools for applications in basic research and translational/clinical fields.

## Introduction

The endometrium represents the dynamic tissue that lines the uterus which is essential for human reproduction, undergoing a menstrual cycle during which an embryo-receptive state is established. The underlying cellular and molecular mechanisms of the reiterative proliferative, secretory and menstrual phases are not well understood, with inaccessibility of these processes *in vivo* impeding in-depth investigation. Moreover, several disorders of the endometrium (such as endometriosis and cancer) cause a significant health burden as well as highly impact fertility, and underlying pathogenic mechanisms remain unclear. Multiple study models have been developed to explore endometrium (patho)biology [extensively reviewed in (1–3)], but show important limitations such as failure to reliably maintain primary cells in long-term culture or their non-physiological nature (e.g. 2D, mouse models, immortalized cell lines). Thus, this research field has long been thwarted by lack of relevant (disease) models.

**Figure 1:**
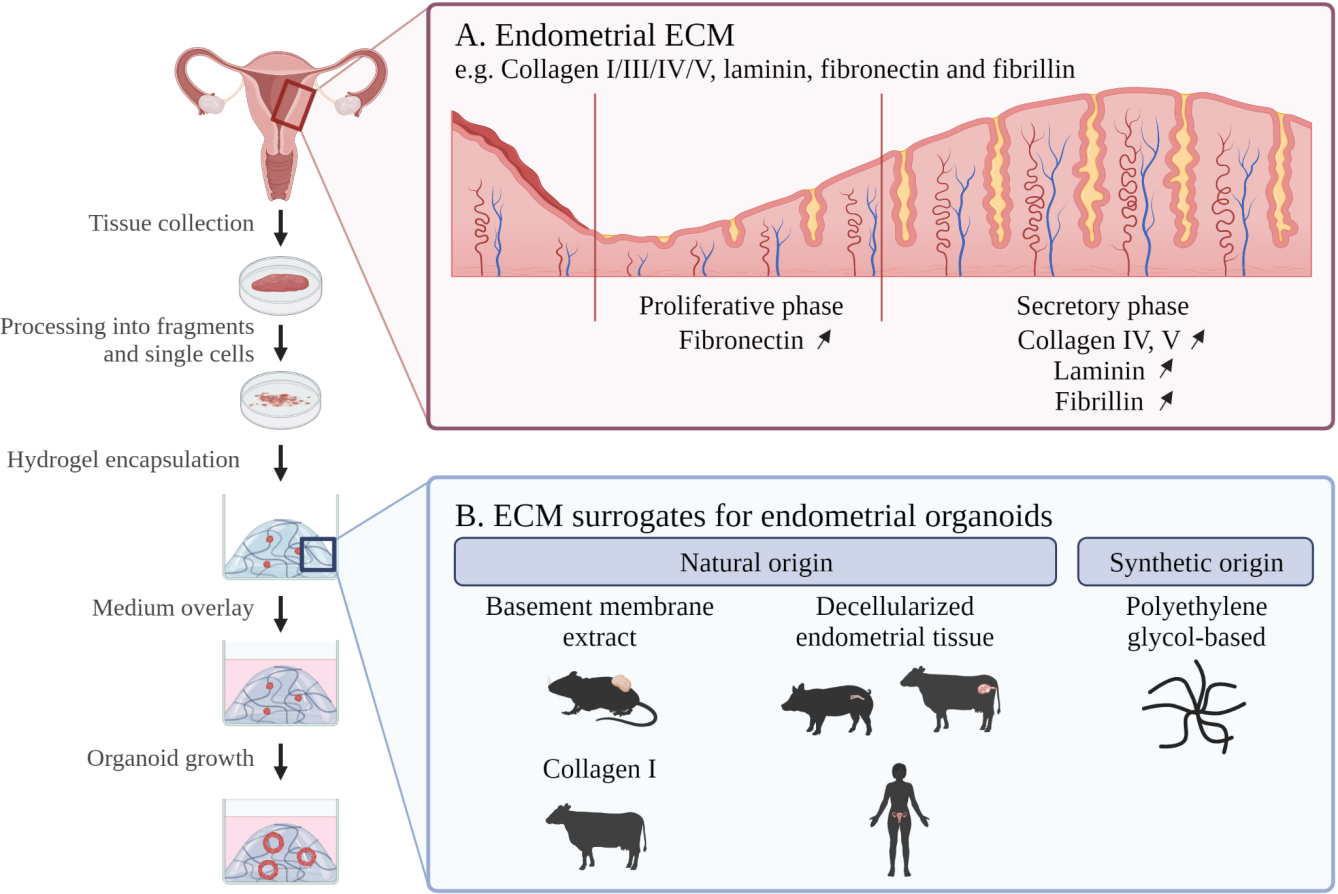
Schematic overview of endometrial organoid culture development with indication of **(A)** *in vivo* endometrial ECM composition and **(B)** ECM scaffolds for *in vitro* endometrial organoid culture. ECM; extracellular matrix. Created with BioRender.com.

Recently, powerful new research models have been established in the form of organoids, shown to be highly instrumental and accurate to study endometrium biology and disease (4–6); and in detail reviewed in (2, 7, 8). Organoids are 3D cell constructions that *in vitro* self-develop from (diseased) tissue (stem) cells when embedded in a supporting ECM (hydrogel) scaffold, and cultured in an optimized, well-defined medium, specifically encompassing stem cell- and embryogenesis-regulatory factors (9) (**Figure 1**). In this review, we will delve into the (dis)advantages of different hydrogel scaffolds utilized to grow endometrial organoids. Hydrogels are polymer-based insoluble networks with a high H_2_0 composition and are divided by, among others, natural or synthetic origin, crosslinking method (physical or covalent bonds) and architecture (fibrous, macro- or nanoporous). Typically, a basement membrane (BM) extract derived from Engelbreth-Holm-Swarm (EHS) mouse sarcoma is used as a substitute for the tissue ECM to grow organoids (10). Commercially available variants include Matrigel, GelTrex and Cultrex BM extract. Matrigel has been the main ECM surrogate used for endometrium organoid culture (4, 5). Despite high level of success, Matrigel has important limitations (summarized in **Table 1** and discussed in detail below), and better ECM-mimicking matrices are actively sought that support endometrial organoid culture as efficiently and reliably. Here, we first describe the characteristics of natural endometrium ECM as ultimate but very challenging benchmark due to its very dynamic ECM composition (throughout the menstrual cycle). Then, we summarize the use of natural hydrogels for endometrium-derived organoid growth and discuss recent attempts to develop more defined matrix scaffolds. Finally, we present challenges and perspectives prevalent in the booming (endometrial) organoid field.

**Table 1:** Overview of the most successful ECM surrogates for endometrial organoid culture.

	Hydrogel or scaffold material	Origin	Main components	Crosslinking method	Advantages	Disadvantages	Main references
Natural	Basement membrane extract (e.g. Matrigel)	Engelbreth-Holm-Swarm mouse sarcoma	• Laminin (~60%) • Collagen IV (~30%) • Entactin (~8%) • Perlecan (~2%) • > 2000 other proteins	Physical and covalent crosslinking (temperature)	• Commercially available • Well-established protocols • Gentle organoid recovery method (temperature)	• Animal- and tumor-derived • Properties cannot be tailored • Batch-to-batch variability • Composition differs from *in vivo* ECM • Undefined ECM composition	(4, 5)
	Endometrial decellularized ECM	Porcine, bovine, human	• Collagen I and III • Elastin • Laminin • Fibronectin • > 1000 other proteins	Covalent crosslinking (ECM fiber self-assembly upon pH neutralization at 37°C)	• Retains biochemical structures of the native ECM • Patient-specific ECM for personalized research • FDA-approved	• Animal-derived (unless human patient-derived) • Batch-to-batch variability • Restricted availability • Undefined ECM composition	(11–13)
	Collagen	Bovine skin	• Collagen I (97%) and III (3%)	Physical and covalent crosslinking (pH and temperature)	• Commercially available • Composed of the most abundant ECM proteins seen in *in vivo* endometrium	• Animal-derived • Properties cannot be tailored • Batch-to-batch variability • Enzymatic (collagenase) recovery method	(14, 15)
Synthetic	Polyethylene glycol (PEG)	PEG	• PEG network • Protease-sensitive crosslinkers (sortase, MMP) • ECM-mimicking peptides (collagen and fibronectin) • Peptides binding cell-secreted basement membrane proteins and fibronectin	Covalent crosslinking (protease-degradable crosslinking peptides)	• Independent tuning of biochemical and biomechanical properties • Defined composition	• Enzymatic recovery method • Need for functionalization with cell-binding peptides • Purely elastic mechanical properties	(16, 17)

## Native endometrium ECM as inspiration for defined organoid matrix development

ECM is the non-cellular network that connects the cellular components of a tissue, thereby establishing biochemical and biomechanical cues that determine key cellular processes such as differentiation, proliferation, homeostasis and migration (18, 19). The desired ECM surrogate for growing endometrium-derived organoids has to mimic the native endometrial ECM as closely as possible, both regarding composition and biomechanical properties.

The endometrial ECM mainly contains collagen IV, fibrillin, laminin and fibronectin (20) (**Table 1**). Importantly, ECM composition is dynamic throughout menstrual cycle and pregnancy (21, 22). For instance, fibronectin becomes more abundant in the proliferative as compared to the secretory phase 1, while laminin, collagen and fibrillin deposition increase in the secretory phase to promote embryo attachment and invasion (23) (**Figure 1**).

Changes in endometrial ECM composition have a direct impact on mechanical properties such as tissue stiffness (24). For example, the nonpregnant endometrium is less stiff than the endometrium of pregnant women (apparent elastic modulus E = ~ 250 Pa and ~ 1250 Pa, respectively) (24). Moreover, ECM composition and structure give rise to complex nonlinear mechanics (19, 25). Among others, endometrial ECM is viscoelastic, meaning that it behaves both as viscous fluid and elastic solid. Viscoelasticity relies on bond remodeling (i.e. breaking and re-forming) between ECM proteins, mainly fibrin and collagen I (19, 26). This continuous remodeling is governed by secreted enzymes such as matrix metalloproteinases (MMPs) (27, 28), and, together with changes in biochemical composition, governs mechanical properties of the ECM. Resultant mechanosensing by the cells through their integrin receptors (e.g. for collagens and fibronectin) leads to activation of intracellular signaling pathways that ultimately influence biological processes such as proliferation and differentiation (19, 25, 29).

Finally, changes in ECM properties have been correlated with endometrial diseases. For example, the ECM remodeling MMPs have been identified as key factors in endometrial cancer [e.g. MMP11 is a type I endometrial cancer marker (30)] as well as in formation and invasion of endometriotic lesions outside the uterus [e.g. high levels of MMP3 (31)]. To understand the relationship between ECM alterations and endometrial (patho-)biology, organoids may provide an excellent tool as the properties of the embedding ECM surrogate can be altered according to the research question.

## Natural hydrogels as supporting scaffold for endometrium-derived organoids

As mentioned, the most used natural hydrogels for organoid culturing, like Matrigel, are BM extracts from EHS mouse sarcoma. Matrigel consists of ~60% laminin (multiple isoforms), ~30% collagen IV, ~8% entactin (glycoprotein molecule bridging laminin and collagen IV), ~2% perlecan (heparin sulfate proteoglycan), and in addition up to 2000 other proteins (32) (**Table 1**). These hydrogels exhibit temperature-dependent gelation, remaining liquid when kept at low temperature (~5°C) and transitioning into a gel-like form above a defined temperature (e.g. 22-37°C for Matrigel). This property enables convenient encapsulation of the cells as well as their retrieval (**Table 1**). Typically, a purification (growth factor-reduced) format is used for organoid culturing to minimize any undesired effects of growth factors present in the BM extract, while factors specifically required for organoid growth are deliberately supplemented to the culture medium.

The first establishment of endometrium-derived organoids occurred in Matrigel (4, 5), further also used by the majority of subsequent endometrial organoid studies (**Table 1**). Indeed, Matrigel was found highly suitable for supporting the development and growth of endometrial organoids across species [i.e. human (4, 5); mouse (4); horse (33)], as well as throughout all menstrual phases in humans (4, 5, 34). Organoids could also recapitulate the different menstrual phases when exposed to the appropriate sex hormones (e.g. mucin production after progesterone treatment) (4, 5). Moreover, Matrigel has been used for both healthy (4, 5) and diseased endometrium, including endometriosis (6, 35, 36), cancer (5, 6) and Mayer-Rokitansky-Küster-Hauser syndrome (37). Importantly, organoid cultures derived from diseased endometrium reproduced key characteristics of the disorder such as mutational landscape (6), morphology (e.g. disorganized epithelium) (5) and gene expression (37). These organoid cultures have been instrumental to investigate endometrial hormonal responses (4, 5, 38), infections [*Chlamydia* (39)], drug screening (6), implantation (14, 40), pathological conditions (6, 37), and regenerative processes (41, 42).

Although highly successful, Matrigel, and generally BM extracts, have important limitations for organoid culturing (43) (**Table 1**). Firstly, their composition is not deeply defined and batch-to-batch variability may result in inconsistent experimental outcomes, although the reproducibility of the endometrial organoid protocol from the initial reports (4, 5) to follow-up studies (6, 14, 35, 38) does not point to major issues in this regard. Nevertheless, better standardization can be achieved by specifying protein concentrations in the BM extracts rather than just the proportion of Matrigel used in the culture (e.g. ‘8 mg/mL protein concentration in Matrigel’ instead of ‘70%’ or ‘1:20 in culture medium’), as protein concentrations can vary between batches and impact gel properties. Secondly, BM extracts do not highly mimic the original tissue’s ECM composition, biochemistry and biomechanics. For instance, Matrigel contains a higher concentration of laminin compared to endometrial ECM, which is rich in collagen fibers (types I and III) and other glycoproteins such as fibronectin (10, 11, 44) (**Table 1**). It is worth noting that endometrial organoids can also produce their own ECM components, with laminin being observed at the outer edge of the organoid structures (6). Thirdly, Matrigel is a very soft viscoelastic hydrogel (E ranging between 70-330 Pa depending on batch and concentration) when compared to endometrium ECM [250-1250 Pa (24)]. Importantly, it is not possible to tune the biochemical cues of Matrigel nor to increase its stiffness to match the *in vivo* situation of (diseased) endometrium. Lastly, the xeno- and tumor-origin of the BM extracts restricts clinical applicability of organoids cultured in these conditions, particularly for applications in regenerative medicine and potentially also drug screening and development (9, 43).

In addition to using these compound BM extracts, endometrial organoids have also been cultured in more pure ECM components (**Table 1**). For example, bovine skin collagen-based hydrogel was applied to co-culture epithelial endometrial cells (as organoids) with the stromal cell component of the tissue (14). These so-called ‘assembloids’ showed gland-like organoids surrounded by stromal cells, and allowed to study epithelial-stromal cell remodeling during embryo interaction (14). Collagen hydrogels, composed of fibrillar networks of collagen I (97%) and III (3%), contain the major collagen types present (and abundant) in natural endometrial ECM (11, 44, 45) (**Table 1**), and present a stiffness (shear modulus G = ~700 Pa) similar to that of native endometrium (14, 24, 46). Despite this fruitful application of collagen gels for endometrial assembloid culture, efficient development and expansion of pure (epithelial) organoids, as needed for extensive downstream analysis, was not reported. Another study developed collagen scaffolds through lyophilization, thereby optimizing pore size to allow efficient exchange of gas and nutrients while still providing sufficient structure (15). Primary endometrial stromal cells were seeded within this collagen scaffold and epithelial cells (derived from organoids but seeded as a 2D monolayer) were grown on top of it (15). The stromal cells were also able to produce collagen themselves (15). Both studies using collagen matrices confirmed the biological functionality of the co-cultures, as supported by hormonal responsiveness (14, 15). Although these collagen hydrogels do not have a tumor origin, they are still animal-derived and suffer from batch-to-batch variability (47). In addition, these hydrogels are not highly tunable regarding biochemical and biomechanical properties, which is needed to match the native tissue.

As the closest *in vivo*-mimicking natural hydrogel for growing endometrium-derived organoids, decellularized ECM (dECM) has been tested (**Table 1**). In one approach, soluble dECM, derived from porcine endometrium, was added to the organoid culture medium with the human endometrial cells (still) embedded in Matrigel, resulting in increased initial proliferation of the cells (12). The medium-dissolved dECM thus provided important biochemical cues. For instance, nicotinamide, previously shown to be essential for endometrial organoid growth (4, 5), was found dispensable in the presence of soluble dECM. In a second approach, cells were embedded in the endometrial dECM-derived hydrogel, obtained from bovine or human origin (11). Decellularization using sodium deoxycholate (SDC) resulted in better preservation of the native ECM structure and composition (such as abundant presence of collagens) than dECM obtained following sodium dodecyl sulfate (SDS) treatment. Moreover, stiffness of SDC-generated dECM hydrogel (storage modulus G’ = 380 Pa) more closely resembled the stiffness of the intact (i.e. before lyophilization and powder milling) decellularized tissue (G’ = 600 Pa) than the hydrogel obtained using SDS treatment (G’ = 70 Pa) or Matrigel (G’ = 75 Pa). The optimized dECM hydrogel, from both bovine and human origin, supported human and mouse endometrium-derived organoid growth comparably to Matrigel. Proteomic analysis put forward that (human) endometrial organoids cultured in SDC-treated dECM hydrogel resembled the patient endometrial tissue better than organoids grown in Matrigel. Moreover, laminin was found to be important for endometrium organoid culturing (as shown for mouse), because addition of bovine-derived laminin to laminin-low (bovine) dECM hydrogel (as obtained using SDS) rescued organoid formation efficiency. Laminin addition also led to a change in morphology from crenelated to rounder structures, more similar to those found in SDC-obtained dECM hydrogel and Matrigel. Taken together, dECM-based matrices, mimicking the *in vivo* ECM better than other natural hydrogels, present a promising tool to enable organoid clinical application. In an alternative approach, organoids were dissociated to single cells which were seeded on top of a human dECM scaffold (13). The organoid-derived cells recellularized both luminal and glandular scaffold surfaces which may provide interesting perspectives for regenerative medicine (13).

Interestingly, dECM-based products have been approved by the US Food and Drug Administration (FDA) (48) which facilitates their clinical translation. However, use of human endometrium-derived dECM will not be straightforward given the limited availability (e.g. sufficient sample material is required if both dECM and organoids need to be derived from the same patient) and the uncertain reproducibility with inter-individual differences as well as intra-patient differences (e.g. along the menstrual cycle). Moreover, obtaining dECM at sufficient purification level may be expensive. Altogether, dECM is not a viable alternative yet for large-scale and long-term growth and expansion of endometrial organoids which is needed for the extensive downstream applications.

Other natural hydrogels have already been applied to develop organoids from several tissues but their application in endometrial organoid culturing remains unexplored. Examples include purified or recombinant ECM components such as fibrin and laminin (49) or hydrogels based on alginate, an FDA-approved polysaccharide derived from brown seaweed, with interesting tunable (visco-)elastic properties (50, 51).

## Synthetic hydrogels as promising scaffolds for endometrium-derived organoid culture

To overcome the above-mentioned shortcomings of natural hydrogels to grow organoids in an efficient, reproducible, xeno-free as well as standardized manner, fully defined synthetic matrix scaffolds are needed and currently actively searched for (52, 53). Precise control of hydrogel composition and independent tuning of mechanical and biochemical properties would be an important advantage over natural matrices. While several synthetic hydrogels have been developed for various tissue organoid models, only one has so far been reported to successfully support the culture of endometrial organoids (16 ^1^; 17) (**Table 1**). The researchers designed a polyethylene glycol (PEG)-based hydrogel that incorporates MMP-sensitive crosslinkers allowing organoid-secreted MMPs to degrade the rigid PEG structure to promote organoid growth. Cleavages sites for the prokaryotic enzyme sortase were also introduced in the PEG gel to enable retrieval of the embedded grown organoids. Moreover, biochemical cues were introduced by adding a synthetic α2β1 or α5β1 integrin-binding peptide based on collagen (GFOGER), which was found critical for successful culture of the endometrial organoids, although they remained smaller compared to those grown in Matrigel (17). Organoids embedded in this synthetic matrix were hormone-responsive with both motile ciliated and secretory epithelial cells present (16). Organoid morphology was dependent on matrix stiffness; stiffer scaffolds (E = ~2,000 Pa and 6,000 Pa) led to crenelated organoids whereas softer matrices (E = ~300 Pa) yielded rounded organoids similar to Matrigel-grown organoids^1^. Addition of a short fibronectin-based synthetic peptide (PHSRN-K-RGD) supported the survival of endometrial stromal cells in a composite epithelial organoid-stromal cell culture (16). Moreover, it improved stromal cell distribution and morphological spreading as compared to PEG-GFOGER alone. This fully defined hydrogel system allowed to co-culture primary endometrial epithelial and stromal cells for two weeks, while natural hydrogels degraded within this timeframe. Such more stable hydrogel would enable to simulate the menstrual cycle *in vitro* for a longer and thus more physiological relevant time period to explore biological and pathological cell interactions and processes (16). As a downside, PEG-based hydrogels consist of flexible networks with only elastic mechanical properties. Viscoelastic semiflexible materials, such as natural hydrogels and primary tissue, exhibit additional important mechanical properties (e.g. stress-stiffening behavior) which can influence epithelial cell growth and patterning (50, 54). The purely elastic PEG-based hydrogels may therefore affect organoids’ cellular and tissue phenotype mimicry differently than natural materials.

The list of synthetic hydrogels, displaying divergent biomechanical properties, is rapidly expanding, thereby providing interesting opportunities toward re-tuned endometrial organoid culture. For example, a light-sensitive synthetic polyvinyl-based hydrogel can guide cell invasion at microscale by *in situ* controlled photopolymerization of the cell-laden hydrogel (55). Such hydrogel could in the future be used to recapitulate ECM remodeling as occurring in both physiological (e.g. menstrual cycle) and pathological conditions (e.g. endometrial cancer and endometriotic lesions) (23, 30, 31). Still other promising hydrogels have been developed, formed by non-covalent interactions such as hydrogen bonds and hydrophobic interactions. Examples are the thermosensitive poly(N-isopropylacrylamide)- (56) and polyisocyanide-based matrices (57). These thermoreversible hydrogels offer the practical advantage over covalently crosslinked hydrogels of allowing gentle cell recovery by simply cooling the sample on ice, similar to the process used with Matrigel which also does not require enzymatic digestion. Hence, these materials will be more user-friendly and facilitate the transition away from Matrigel.

## Challenges and future perspectives

Endometrium-derived organoid models provide powerful *in vitro* tools to decipher endometrial biology and disease. To date, 3D organoids are mainly grown in BM extract (typically Matrigel), a one-size-fits-all ECM surrogate which enables efficient organoid development and growth from various tissues, in both healthy and diseased conditions. However, this BM extract poses several challenges, particularly its poor definition, animal and tumor origin. Standardization and clinical translation urge for a better (fully) defined matrix which can support the efficient growth of organoids while reliably recapitulating the characteristics of the original tissue epithelium. Along this line, biochemical and biomechanical properties of the native tissue should be recapitulated as closely as possible. Although more defined ECM component matrices (such as collagen hydrogels) or endometrial dECM-based gels may be a step forward, they still suffer from batch-to-batch variability and mostly non-human origin. Thus, developing fully synthetic and tunable biomimetic hydrogels is necessary to tailor the matrix to the *in vivo* counterpart, encompassing important parameters such as biocompatibility, stiffness, viscoelasticity and porosity. The PEG-based hydrogel discussed above provides a first step but still lacks important biomechanical properties and the fiber architecture of the *in vivo* tissue ECM. New synthetic hydrogels, fully defined and tunable, are needed to advance standardized (while still efficient) organoid culturing from endometrial epithelium. Such better-defined hydrogels will also enable to investigate the effect of individually added ECM components on organoid formation, growth and phenotype, and on how ECM alterations influence endometrial cell behavior (e.g. pathology, response to hormones). A further challenge includes the simulation of the dynamic endometrial ECM composition and remodeling as occurring throughout the menstrual cycle and in pathological conditions. Hydrogels that provide spatial manipulation during organoid growth can offer a solution. Another challenge will be to identify defined matrices for further advanced mimicry of the endometrium, i.e. that support the reliable growth and culture of not only the epithelial cells but also other components of the endometrial tissue such as stromal, endothelial and immune cells. The co-culture of epithelial and stromal cells in assembloids, as achieved in collagen and PEG-based matrices, provides a first step toward this ambitious goal. Also immune cells and associated inflammatory processes play an important role in the endometrial processes of menstruation (58) and implantation (59), as well as in pathologies (e.g. endometriosis) (59). Including immune cells into organoid cultures would be another important advancement in endometrial 3D modeling. To study inflammatory processes, immunomodulatory factors (cytokines) are often supplemented to the organoid medium to mimic the presence of immune cells. One study reported the co-culture of endometrial organoids and primary bone marrow-derived neutrophils to study the primary immune response after *Chlamydia* infection (60). *Chlamydia*-secreted effectors restricted neutrophil recruitment to infected organoids (60). Co-culture with other immune cell types present in the endometrium, such as macrophages and uterine natural killer cells, is needed for further enhancing the physiological relevance of the models and enables additional research in innate and adaptive immune responses (58). Interesting evolutions within the tissue engineering field can also be applied to better reconstitute the complex make-up and architecture of the endometrium, including bioprinting approaches (although still very challenging) in which appropriate hydrogels are used as bio-ink, and microfluidic technologies containing different endometrial cell types in interconnected microchambers to recapitulate cell-cell and cell-matrix interplay (1). As an asset, different types of hydrogels, each best fitting a specific cell type, can be used in the individual chambers, thereby avoiding the need for a single hydrogel that fits all cell types.

In conclusion, the identification and application of more defined hydrogels will allow to advance endometrial organoid modeling toward more reproducible and standardized applications including genetic and drug screenings. The current development of a range of defined hydrogels can provide researchers to choose the most fitting matrix to answer their research question into endometrium (patho)biology.

## Author contributions

SDV and HV designed the review. SDV and CMC collected all the information and wrote the manuscript. HV and SR co-wrote and critically revised the manuscript, and HV finalized it. All authors contributed to the article and approved the submitted version.

## Footnotes

1 Not yet published following completed peer review and thus still to be interpreted with caution.

## References

[1] Murphy AR Campo H Kim JJ. Strategies for modelling endometrial diseases. Nat Rev Endocrinol (2022) 18:727–43. doi: 10.1038/S41574-022-00725-Z PMC10052865 36050476

[2] Maenhoudt N De Moor A Vankelecom H. Modeling endometrium biology and disease. J Pers Med (2022) 12:1048. doi: 10.3390/jpm12071048 35887546 PMC9316888

[3] Francés-Herrero E Lopez R Hellström M de Miguel-Gómez L Herraiz S Brännström M. Bioengineering trends in female reproduction: a systematic review. Front Bioeng Biotechnol (2022) 28:1–40. doi: 10.1093/humupd/dmac025 PMC9629485 35652272

[4] Boretto M Cox B Noben M Hendriks N Fassbender A Roose H. Development of organoids from mouse and human endometrium showing endometrial epithelium physiology and long-term expandability. Development (2017) 144:1775–86. doi: 10.1242/dev.148478 28442471

[5] Turco MY Gardner L Hughes J Cindrova-Davies T Gomez MJ Farrell L. Long-term, hormone-responsive organoid cultures of human endometrium in a chemically defined medium. Nat Cell Biol (2017) 19:568–77. doi: 10.1038/ncb3516 PMC5410172 28394884

[6] Boretto M Maenhoudt N Luo X Hennes A Boeckx B Bui B. Patient-derived organoids from endometrial disease capture clinical heterogeneity and are amenable to drug screening. Nat Cell Biol (2019) 21:1041–51. doi: 10.1038/s41556-019-0360-z 31371824

[7] Song Y Fazleabas AT. Endometrial organoids: a rising star for research on endometrial development and associated diseases. Reprod Sci (2021) 28:1626–36. doi: 10.1007/s43032-021-00471-z 33533008

[8] Lou L Kong S Sun Y Zhang Z Wang H. Human endometrial organoids: recent research progress and potential applications. Front Cell Dev Biol (2022) 10:844623. doi: 10.3389/fcell.2022.844623 35242764 PMC8885623

[9] Schutgens F Clevers H. Human organoids: tools for understanding biology and treating diseases. Annu Rev Pathol Mech Dis (2020) 15:211–34. doi: 10.1146/annurev-pathmechdis-012419-032611 31550983

[10] Kleinman HK Martin GR. Matrigel: basement membrane matrix with biological activity. Semin Cancer Biol (2005) 15:378–86. doi: 10.1016/j.semcancer.2005.05.004 15975825

[11] Jamaluddin MFB Ghosh A Ingle A Mohammed R Ali A Bahrami M. Bovine and human endometrium-derived hydrogels support organoid culture from healthy and cancerous tissues. Proc Natl Acad Sci USA (2022) 119:e2208040119. doi: 10.1073/pnas.2208040119 36279452 PMC9636948

[12] Francés-Herrero E Juárez-Barber E Campo H López-Martínez S de Miguel-Gómez L Faus A. Improved models of human endometrial organoids based on hydrogels from decellularized endometrium. J Pers Med (2021) 11:504. doi: 10.3390/jpm11060504 34205034 PMC8229407

[13] Venkata VD Jamaluddin MFB Goad J Drury HR Tadros MA Lim R. Development and characterization of human fetal female reproductive tract organoids to understand Müllerian duct anoMalies. Proc Natl Acad Sci USA (2022) 119:e2118054119. doi: 10.1073/pnas.2118054119 35858415 PMC9335258

[14] Rawlings TM Makwana K Taylor DM Molè MA Fishwick KJ Tryfonos M. Modelling the impact of decidual senescence on embryo implantation in human endometrial assembloids. Elife (2021) 10:e69603. doi: 10.7554/eLife.69603 34487490 PMC8523170

[15] Abbas Y Brunel LG Hollinshead MS Fernando RC Gardner L Duncan I. Generation of a three-dimensional collagen scaffold-based model of the human endometrium. Interface Focus (2020) 10:20190079. doi: 10.1098/rsfs.2019.0079 32194932 PMC7061944

[16] Gnecco JS Brown A Buttrey K Ives C Brittany A Baugh L. Organoid coculture model of the cycling human endometrium in a fully-defined synthetic extracellular matrix reveals epithelial-stromal crosstalk. [Preprint] (2022) Available at: doi: 10.1101/2021.09.30.462577

[17] Hernandez-Gordillo V Kassis T Lampejo A Choi GH Gamboa ME Gnecco JS. Fully synthetic matrices for in vitro culture of primary human intestinal enteroids and endometrial organoids. Biomaterials (2020) 254:120125. doi: 10.1016/j.biomaterials.2020.120125 32502894 PMC8005336

[18] Bonnans C Chou J Werb Z. Remodelling the extracellular matrix in development and disease. Nat Rev Mol Cell Biol (2014) 15:786–801. doi: 10.1038/nrm3904 25415508 PMC4316204

[19] Chaudhuri O Cooper-White J Janmey PA Mooney DJ Shenoy VB. Effects of extracellular matrix viscoelasticity on cellular behaviour. Nature (2020) 584:535–46. doi: 10.1038/s41586-020-2612-2 PMC7676152 32848221

[20] Olalekan SA Burdette JE Getsios S Woodruff TK Kim JJ. Development of a novel human recellularized endometrium that responds to a 28-day hormone treatment. Biol Reprod (2017) 96:971–81. doi: 10.1093/biolre/iox039 PMC5803758 28449068

[21] Iwahashi M Muragaki Y Ooshima A Yamoto M Nakano R. Alterations in distribution and composition of the extracellular matrix during decidualization of the human endometrium. J Reprod Fertil (1996) 108:147–55. doi: 10.1530/JRF.0.1080147 8958841

[22] Harrington DJ Lessey BA Rai V Bergqvist A Kennedy S Manek S. Tenascin is differentially expressed in endometrium and endometriosis. J Pathol (1999) 187:242–8. doi: 10.1002/(SICI)1096-9896(199901)187:2<242::AID-PATH221>3.0.CO;2-T 10365101

[23] O’Connor BB Pope BD Peters MM Ris-Stalpers C Parker KK. The role of extracellular matrix in normal and pathological pregnancy: future applications of microphysiological systems in reproductive medicine. Exp Biol Med (2020) 245:1163. doi: 10.1177/1535370220938741 PMC7400725 32640894

[24] Abbas Y Carnicer-Lombarte A Gardner L Thomas J Brosens JJ Moffett A. Tissue stiffness at the human maternal–fetal interface. Hum Reprod (2019) 34:1999–2008. doi: 10.1093/humrep/dez139 31579915 PMC6809602

[25] Saraswathibhatla A Indana D Chaudhuri O. Cell–extracellular matrix mechanotransduction in 3D. Nat Rev Mol Cell Biol (2023) 24:495–516. doi: 10.1038/s41580-023-00583-1 PMC10656994 36849594

[26] Münster S Jawerth LM Leslie BA Weitz JI Fabry B Weitz DA. Strain history dependence of the nonlinear stress response of fibrin and collagen networks. Proc Natl Acad Sci USA (2013) 110:12197–202. doi: 10.1073/pnas.1222787110 PMC3725119 23754380

[27] Stamenkovic I. Extracellular matrix remodelling: the role of matrix metalloproteinases. J Pathol (2003) 200:448–64. doi: 10.1002/path.1400 12845612

[28] Padežnik T Oleksy A Cokan A Takač I Sobočan M. Changes in the extracellular matrix in endometrial and cervical cancer: a systematic review. Int J Mol Sci (2023) 24:5463. doi: 10.3390/ijms24065463 36982551 PMC10052846

[29] Kechagia JZ Ivaska J Roca-Cusachs P. Integrins as biomechanical sensors of the microenvironment. Nat Rev Mol Cell Biol (2019) 20:457–73. doi: 10.1038/s41580-019-0134-2 31182865

[30] Gómez-Macías GS Garza-Rodríguez ML Garza-Guajardo R Monsiváis-Ovalle D Ancer-Rodríguez J Barrera-Saldaña HA. Overexpression of the matrix metalloproteinase 11 gene is a potential biomarker for type 1 endometrial cancer. Oncol Lett (2018) 16:1073–8. doi: 10.3892/ol.2018.8714 PMC6019964 29963184

[31] Luddi A Marrocco C Governini L Semplici B Pavone V Luisi S. Expression of matrix metalloproteinases and their inhibitors in endometrium: high levels in endometriotic lesions. Int J Mol Sci (2020) 21:2840. doi: 10.3390/IJMS21082840 32325785 PMC7215833

[32] Hughes CS Postovit LM Lajoie GA. Matrigel: a complex protein mixture required for optimal growth of cell culture. Proteomics (2010) 10:1886–90. doi: 10.1002/pmic.200900758 20162561

[33] Thompson RE Johnson AK Dini P Turco MY Prado TM Premanandan C. Hormone-responsive organoids from domestic mare and endangered Przewalski’s horse endometrium. Reproduction (2020) 160:819–31. doi: 10.1530/REP-20-0266 33112764

[34] Cindrova-Davies T Zhao X Elder K Jones CJP Moffett A Burton GJ. Menstrual flow as a non-invasive source of endometrial organoids. Commun Biol (2021) 4:651. doi: 10.1038/s42003-021-02194-y 34140633 PMC8211845

[35] Luddi A Pavone V Semplici B Governini L Criscuoli M Paccagnini E. Organoids of human endometrium: a powerful in *vitro* model for the endometrium-embryo cross-talk at the implantation site. Cells (2020) 9:1121. doi: 10.3390/cells9051121 32366044 PMC7291023

[36] Tan Y Flynn WF Sivajothi S Luo D Bozal SB Davé M. Single-cell analysis of endometriosis reveals a coordinated transcriptional programme driving immunotolerance and angiogenesis across eutopic and ectopic tissues. Nat Cell Biol (2022) 24:1306–18. doi: 10.1038/s41556-022-00961-5 PMC9901845 35864314

[37] Brucker SY Hentrich T Schulze-Hentrich JM Pietzsch M Wajngarten N Singh AR. Endometrial organoids derived from Mayer–Rokitansky–Küster–Hauser syndrome patients provide insights into disease-causing pathways. Dis Model Mech (2022) 15:dmm049379. doi: 10.1242/dmm.049379 35394036 PMC9118093

[38] Garcia-Alonso L Handfield LF Roberts K Nikolakopoulou K Fernando RC Gardner L. Mapping the temporal and spatial dynamics of the human endometrium in *vivo* and in *vitro* . Nat Genet (2021) 53:1698–711. doi: 10.1038/s41588-021-00972-2 PMC8648563 34857954

[39] Bishop RC Boretto M Rutkowski MR Vankelecom H Derré I. Murine endometrial organoids to model Chlamydia infection. Front Cell Infect Microbiol (2020) 10:416. doi: 10.3389/fcimb.2020.00416 32923409 PMC7456808

[40] Kagawa H Javali A Khoei HH Sommer TM Sestini G Novatchkova M. Human blastoids model blastocyst development and implantation. Nature (2022) 601:600–5. doi: 10.1038/s41586-021-04267-8 PMC8791832 34856602

[41] Gebril M Aboelmaaty A Al Balah O Taha T Abbassy A Elnoury MAH. Bio-modulated mice epithelial endometrial organoids by low-level laser therapy serves as an in *vitro* model for endometrial regeneration. Reprod Biol (2021) 21:100564. doi: 10.1016/j.repbio.2021.100564 34662815

[42] Zhang H Xu D Li Y Lan J Zhu Y Cao J. Organoid transplantation can improve reproductive prognosis by promoting endometrial repair in mice. Int J Biol Sci (2022) 18:2627–38. doi: 10.7150/ijbs.69410 PMC8990468 35414792

[43] Aisenbrey EA Murphy WL. Synthetic alternatives to matrigel. Nat Rev Mater (2020) 5:539–51. doi: 10.1038/s41578-020-0199-8 PMC7500703 32953138

[44] López-Martínez S Campo H de Miguel-Gómez L Faus A Navarro AT Díaz A. A natural xenogeneic endometrial extracellular matrix hydrogel toward improving current human in *vitro* models and future in *vivo* applications. Front Bioeng Biotechnol (2021) 9:639688. doi: 10.3389/fbioe.2021.639688 33748086 PMC7973233

[45] Oefner CM Sharkey A Gardner L Critchley H Oyen M Moffett A. Collagen type IV at the fetal–maternal interface. Placenta (2015) 36:59–68. doi: 10.1016/j.placenta.2014.10.012 25465704 PMC4302218

[46] Bagley B. Advanced biomatrix. Collagen gelation kinetics and shear modulus (2019). Available at: https://advancedbiomatrix.com/3d-collagen-hydrogel-stiffness.html.

[47] Antoine EE Vlachos PP Rylander MN. Review of collagen I hydrogels for bioengineered tissue microenvironments: characterization of mechanics, structure, and transport. Tissue Eng Part B Rev (2014) 20:683–96. doi: 10.1089/ten.teb.2014.0086 PMC4241868 24923709

[48] Saldin LT Cramer MC Velankar SS White LJ Badylak SF. Extracellular matrix hydrogels from decellularized tissues: structure and function. Acta Biomater (2017) 49:1–15. doi: 10.1016/j.actbio.2016.11.068 27915024 PMC5253110

[49] Broguiere N Isenmann L Hirt C Ringel T Placzek S Cavalli E. Growth of epithelial organoids in a defined hydrogel. Adv Mater (2018) 30:1801621. doi: 10.1002/adma.201801621 30203567

[50] Elosegui-Artola A Gupta A Najibi AJ Seo BR Garry R Darnell M. Matrix viscoelasticity controls spatio-temporal tissue organization. Nat Mater (2023) 22:117–27. doi: 10.1038/s41563-022-01400-4 PMC10332325 36456871

[51] Cattelan G Guerrero Gerbolés A Foresti R Pramstaller PP Rossini A Miragoli M. Alginate formulations: current developments in the race for hydrogel-based cardiac regeneration. Front Bioeng Biotechnol (2020) 8:414. doi: 10.3389/fbioe.2020.00414 32457887 PMC7226066

[52] Gjorevski N Sachs N Manfrin A Giger S Bragina ME Ordóñez-Morán P. Designer matrices for intestinal stem cell and organoid culture. Nature (2016) 539:560–4. doi: 10.1038/nature20168 27851739

[53] Below CR Kelly J Brown A Humphries JD Hutton C Xu J. A microenvironment-inspired synthetic three-dimensional model for pancreatic ductal adenocarcinoma organoids. Nat Mater (2022) 21:110–9. doi: 10.1038/s41563-021-01085-1 PMC7612137 34518665

[54] Vernerey FJ Sridhar SL Muralidharan A Bryant SJ. Mechanics of 3D cell–hydrogel interactions: experiments, models, and mechanisms. Chem Rev (2021) 121:11085–148. doi: 10.1021/acs.chemrev.1c00046 34473466

[55] Qin XH Wang X Rottmar M Nelson BJ Maniura-Weber K. Near-infrared light-sensitive polyvinyl alcohol hydrogel photoresist for spatiotemporal control of cell-instructive 3D microenvironments. Adv Mater (2018) 30:1705564. doi: 10.1002/adma.201705564 29333748

[56] Johnson HJ Chakraborty S Muckom RJ Balsara NP Schaffer DV. A scalable and tunable thermoreversible polymer for 3D human pluripotent stem cell biOmanufacturing. iScience (2022) 25:104971. doi: 10.1016/j.isci.2022.104971 36147944 PMC9485071

[57] Zhang Y Tang C Span PN Rowan AE Aalders TW Schalken JA. Polyisocyanide hydrogels as a tunable platform for mammary gland organoid formation. Adv Sci (2020) 7:2001797. doi: 10.1002/advs.202001797 PMC7509700 32999851

[58] Critchley HOD Maybin JA Armstrong GM Williams ARW. Physiology of the endometrium and regulation of menstruation. Physiol Rev (2020) 100:1149–79. doi: 10.1152/physrev.00031.2019 32031903

[59] Sieg W Kiewisz J Podolak A Jakiel G Woclawek-Potocka I Lukaszuk J. Inflammation-related molecules at the maternal-fetal interface during pregnancy and in pathologically altered endometrium. Curr Issues Mol Biol (2022) 44:3792–808. doi: 10.3390/cimb44090260 PMC9497515 36135172

[60] Dolat L Valdivia RH. An endometrial organoid model of interactions between Chlamydia and epithelial and immune cells. J Cell Sci (2021) 134:jcs252403. doi: 10.1242/jcs.252403 33468625 PMC7970307

